# The Effect of Three-Dimensional Preoperative Simulation on Liver Surgery

**DOI:** 10.1007/s00268-017-3933-7

**Published:** 2017-03-07

**Authors:** Ken Nakayama, Yukio Oshiro, Ryoichi Miyamoto, Keisuke Kohno, Kiyoshi Fukunaga, Nobuhiro Ohkohchi

**Affiliations:** Department of Gastrointestinal and Hepatobiliary–Pancreatic Surgery, Faculty of Medicine, University of Tsukuba, Tsukuba, Ibaraki 305-8575 Japan

## Abstract

**Background:**

In the past decade, three-dimensional (3D) simulation has been commonly used for liver surgery. However, few studies have analyzed the usefulness of this 3D simulation. The aim of this study was to evaluate the effect of 3D simulation on the outcome of liver surgery.

**Methods:**

We retrospectively analyzed 240 consecutive patients who underwent liver resection. The patients were divided into two groups: those who received 3D preoperative simulation (“3D group”, *n* = 120) and those who did not undergo 3D preoperative simulation (“without 3D group”, *n* = 120). The perioperative outcomes, including operation time, blood loss, maximum aspartate transaminase level, length of postoperative stay, postoperative complications and postoperative mortality, were compared between the two groups. The predicted resected liver volume was compared with the actual resected volume.

**Results:**

The median operation time for the 3D group was 36 min shorter than that for the without 3D group (*P* = 0.048). There were no significant differences in other outcomes between the two groups. A subgroup analysis revealed that the operation time of repeated hepatectomy and segmentectomy for the 3D group was shorter than that for the without 3D group (*P* = 0.03). There was a strong correlation between the predicted liver volume and the actual resected liver weight (*r* = 0.80, *P* < 0.001).

**Conclusion:**

These findings demonstrate that 3D preoperative simulation may reduce the operation time, particularly for repeated hepatectomy and segmentectomy.

## Introduction

The liver contains a highly complex vascular system. To remove liver tumors safely and reliably, surgeons must recognize the positional relationship between the blood vessels of the liver and the tumor. Furthermore, the volume of the remaining liver must be assessed for safe liver resection, particularly for patients with impaired liver function. Three-dimensional (3D) simulation software aids in determining the positional relationships of vessels and in calculating liver volume. Recently, 3D simulation in liver surgery has become common in Japan because it is covered by insurance. Researchers have reported the use of 3D simulation for anatomic analysis, liver volume estimation, irrigation area and procedural planning [1]. However, the clinical efficacy of 3D simulation in terms of perioperative outcomes has not yet been analyzed [2]. The aim of this study was to evaluate the effect of 3D simulation on liver surgery.

## Methods

### Patients

**Fig. 1:**
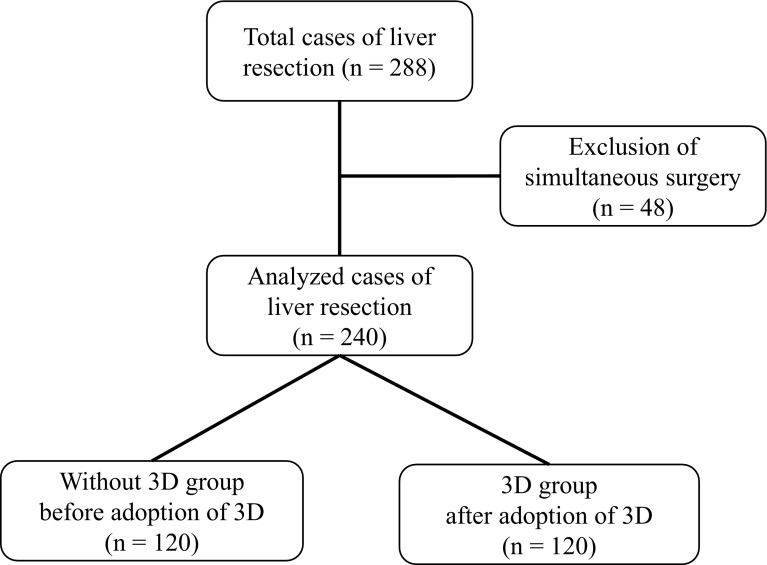
Flowchart of the study. Of the 288 consecutive patients who underwent liver resection, 48 patients who had received a simultaneous surgery were excluded. The remaining 240 patients were divided into two groups: before adoption of 3D preoperative simulation (the without 3D group) or after adoption of 3D preoperative simulation (the 3D group)

Between July 2006 and September 2013, a total of 288 patients underwent liver resection at the University of Tsukuba Hospital. Of these patients, 48 who underwent liver resection and another surgery simultaneously were excluded. The remaining 240 patients were analyzed. These patients included 168 males and 72 females with a median age of 65 years (range 17–81 years). Patients were sequentially divided into two groups: those without 3D preoperative simulation (without 3D group, *n* = 120) and those with 3D preoperative simulation (3D group, *n* = 120) (Fig. 1). Before 2010 (and thus prior to the adoption of the 3D simulation system), 120 sequential patients underwent a hepatectomy without preoperative 3D simulation (Table 1). These patients included 79 men and 41 women with a median age of 65 years (range 22–80 years). The preoperative diagnoses for these patients were as follows: 59 diagnosis of hepatocellular carcinoma (HCC), 3 of intrahepatic cholangiocarcinoma (ICC), 10 of extrahepatic cholangiocarcinoma, 29 of metastatic liver tumors, 7 of donor for liver transplantation, and 9 of other. The patients were classified according to the Child–Pugh classification as grade A (*n* = 111), grade B (*n* = 8) or grade C (*n* = 1). One case received preoperative portal vein embolization (PVE) to enlarge the remnant liver volume. Twenty-nine patients had a history of hepatectomy. The surgical procedure performed depended on the location of the primary tumor and liver function. Ten patients underwent extended left hepatectomy. Five patients underwent extended right hepatectomy. Fourteen patients underwent left hepatectomy. Four patients were subjected to right hepatectomy. Twenty-one patients were subjected to sectionectomy. Twenty patients underwent segmentectomy, and 46 patients underwent partial resection. The characteristics of the 120 patients for whom preoperative simulation was performed are detailed in Table 1. These 120 patients consisted of 89 men and 31 women with a median age of 67 years (range 17–81 years). Of these patients, 62 were preoperatively diagnosed with HCC, 15 with ICC, 10 with extrahepatic cholangiocarcinoma, 24 with metastatic liver tumors, 3 as a transplantation donor, and 6 with other. The patients were classified according to the Child–Pugh classification as grade A (*n* = 115), grade B (*n* = 4) or grade C (*n* = 1). Four cases received preoperative PVE. Nine patients had a history of hepatectomy. Fourteen patients underwent extended left hepatectomy. Seven patients were subjected to extended right hepatectomy. Eleven patients underwent left hepatectomy, whereas three underwent right hepatectomy. Twenty-four patients underwent sectionectomy, and 12 underwent segmentectomy. Forty-nine patients experienced partial resection. There were no significant differences in characteristics other than the length of the surgeon’s career between the without 3D group and the 3D group.

**Table 1:** Patient characteristics

Characteristics	Without 3D group	3D group	*P* value
	*n* = 120	*n* = 120	
*Age* (*years*)			
Median (range)	65 (22–80)	67 (17–81)	0.30
*Sex*			
Male (%)	79 (65.8)	89 (74.2)	0.16
Female (%)	41 (34.2)	31 (25.8)	
*Diagnosis*			
Hepatocellular carcinoma	59	62	0.15
Intrahepatic cholangiocarcinoma	3	15	
Extrahepatic cholangiocarcinoma	10	10	
Metastatic tumor	29	24	
Transplantation donor	7	3	
Other	9	6	
*Liver function*			
Child–Pugh A	111	115	0.52
Child–Pugh B	8	4	
Child–Pugh C	1	1	
Preoperative portal vein embolization	1	4	0.37
Previous hepatectomy	29	9	<0.01
*Procedure*			
Extended left hepatectomy^†^	10	14	0.89
Extended right hepatectomy^††^	5	7	
Left hepatectomy	14	11	
Right hepatectomy	4	3	
Sectionectomy	21	24	
Segmentectomy	20	12	
Partial resection	46	49	
*Surgeon career length* (*year*)			
Median (range)	18 (4–32)	11.5 (3–33)	<0.01

All the values, except for patient age and surgeon career length, are expressed as the number of patients

^†^Includes left trisectionectomy

^††^Includes right trisectionectomy

### Computed tomography

Computed tomography (CT) images were acquired using an IDT-16 imager (Royal Philips, Eindhoven, the Netherlands). The scan settings included a pitch of 17, a 0.75 s scan time per rotation, a table speed of 12 mm/rotation and a detector configuration of 0.75 × 16 mm. A power injector was used to administer 100 mL of iopamidol (370 mg/mL) at 4 mL/s through a 20-G high-pressure intravenous catheter. The CT protocol obtained three sets of hepatic images in succession, including images of the arterial, portal venous and hepatic venous phases. The arterial phase images were obtained 5 s after peak aortic enhancement. The portal venous phase images were acquired 70 s after the start of the injection. The hepatic venous phase was obtained 180 s after the start of the injection.

### Preoperative liver surgery simulation

**Fig. 2:**
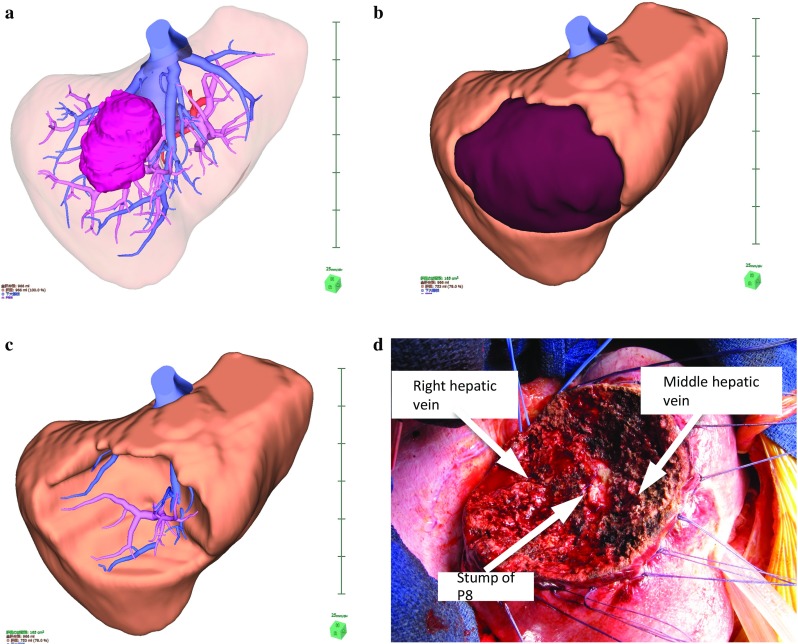
A case of segment 8 segmentectomy. A 3D image was generated from patient CT DICOM data using a 3D image analysis system. A large tumor located in segment 8 of the liver is shown (**a**). S8 segmentectomy was planned, and the resection line was drawn along the demarcation line of P8 (**b**). An image of the resected liver (**c**). The position of the stump of P8 and the running directions of the middle hepatic vein and the right hepatic vein were similar to those determined in the preoperative simulation (**d**)

The two-dimensional (2D) CT patient images were transferred to a workstation (SYNAPSE VINCENT: FUJIFILM Medical Co., Ltd., Tokyo, Japan), and the authors (Y. O. and K.N.) generated the 3D images. We were able to perform a virtual hepatectomy in a preoperative operation meeting with the surgical team. The choice of resection was made based on tumor size, tumor location, liver function (ICGR 15, Child–Pugh classification) and surgical margins as determined by virtual hepatectomy (Fig. 2). We brought these 3D images of the liver into the operating room, and the surgical team could view the simulation images on a large-screen display during the actual hepatectomy.

### Liver resection

All liver resections were performed with an ultrasonic aspirator. Essentially, we carried out intraoperative ultrasonography (US) consistently between the two periods. Almost all patients underwent liver resection via the total Pringle maneuver. Fundamentally, total Pringle was performed by clamping the hepatoduodenal ligament for 15 min and releasing it for 5 min. An anatomic segmentectomy was performed as follows: After the objective portal vein was revealed, a test clamp was performed to recognize the demarcation area, which confirmed the correct resection line. The puncture and staining techniques are rarely performed. These procedures were carried out identically in the two groups.

### Perioperative outcomes

Various perioperative outcomes, including operation time, blood loss, maximum aspartate transaminase (AST) level, length of postoperative stay and postoperative mortality within 30 and 90 days, were compared between the two groups. Postoperative complications were assessed by the Clavien–Dindo classification [3], and complications of grade IIIa or higher were included. Postoperative liver failure and postoperative bile leakage were defined and graded based on the classification system of the International Study Group of Liver Surgery (ISGLS), and grade B or C complications were included [4, 5].

### Validation of 3D simulation

We validated the predicted resected liver volume by comparison with the actual resected volume. 1 gram of liver tissue was assumed equal to a volume of 1 mL [6–9].

### Statistical analysis

Continuous data were expressed as the median (range) using box and whisker plots and were compared using the Mann–Whitney *U* test. Categorical data were compared using the Chi-square test or Fisher’s exact test as appropriate. Correlations were visualized in scatterplots and were analyzed using Spearman’s test. Differences among three or more independent groups were compared using the Kruskal–Wallis test (IBM SPSS Statistics 22.0, IBM Corp., NY, USA). *P* values <0.05 were considered to be statistically significant.

## Results

### Perioperative outcomes

The perioperative outcomes, including operation time, blood loss, maximum AST level, length of postoperative stay, postoperative complications and postoperative mortality, are summarized in Table 2. The median operation time was 36 min shorter for the 3D group than for the without 3D group (*P* = 0.048). No significant differences were observed in intraoperative blood loss, length of postoperative stay, postoperative complications or postoperative mortality between the two groups.

**Table 2:** Perioperative outcomes

Perioperative outcome	Without 3D group	3D group	*P* value
	*n* = 120	*n* = 120	
Operation time (min)*	373 (125–866)	337 (92–860)	0.048
Blood loss (mL)*	550 (50–5840)	597 (18–16,060)	0.26
Maximum AST level (IU/L)*	389 (67–4385)	452 (58–3129)	0.10
Postoperative stay (days)*	12 (5–75)	12 (6–100)	0.39
Postoperative complication^†^ (case)**	14	13	0.84
Postoperative liver failure^††^ (case)**	5	14	0.06
Postoperative bile leakage^††^ (case)**	7	7	0.78
Postoperative mortality 30 days (case)**	1	1	1.0
Postoperative mortality 90 days (case)**	3	2	1.0

* values are expressed as the median (range)

** values are expressed as the number of patients

^†^Clavien–Dindo grade IIIa or higher

^††^ISGLS grade B or higher

**Fig. 3:**
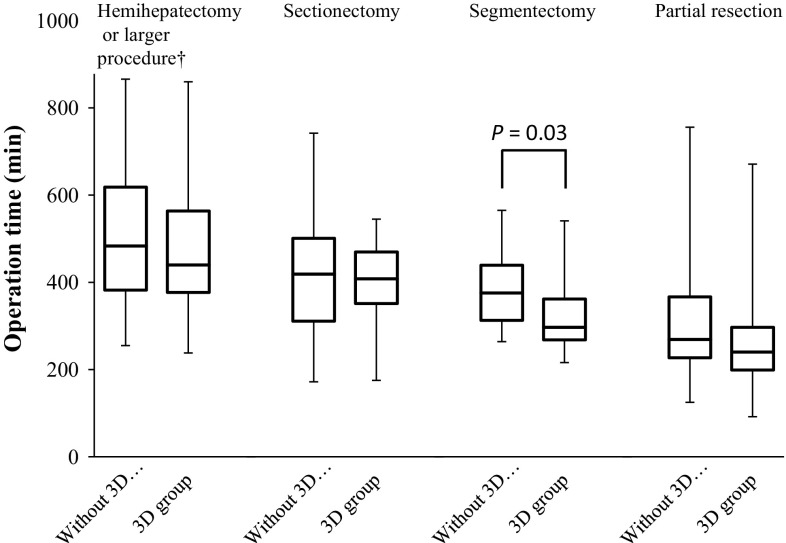
Subgroup analysis of operation time. In the procedure subgroups, the operation time was shorter for the 3D group than for the without 3D group for segmentectomy (*P* = 0.03)

Subsequently, we performed a subgroup analysis of the operation time (Table 3). In the subgroup with previous hepatectomy, the operation time for repeated hepatectomy was shorter in the 3D group than that in the without 3D group (*P* = 0.03). In the procedure subgroups, the operation time for segmentectomy was shorter in the 3D group than that in the without 3D group (*P* = 0.03) (Fig. 3).

**Table 3:** Subgroup analysis of operation time

Subgroup	Operation time (min)		*P* value
	Without 3D group	3D group	
*Previous hepatectomy*			
No	379 (125–868)	346 (110–860)	0.05
Yes	340 (157–760)	210 (92–789)	0.03
*Procedure*			
Hemihepatectomy or more complicated^†^	483.5 (255–866)	440 (238–860)	0.21
Sectionectomy	419 (172–742)	402.5 (175–545)	0.29
Segmentectomy	375.5 (264–565)	332 (175–545)	0.03
Partial resection	269 (125–756)	240 (92–671)	0.06

Values are expressed as the median (range)

^† ^hemihepatectomy or more complicated includes extended left (right) hepatectomy and left (right) hepatectomy

### Validation of resected liver volume and predicted resected liver area

**Fig. 4:**
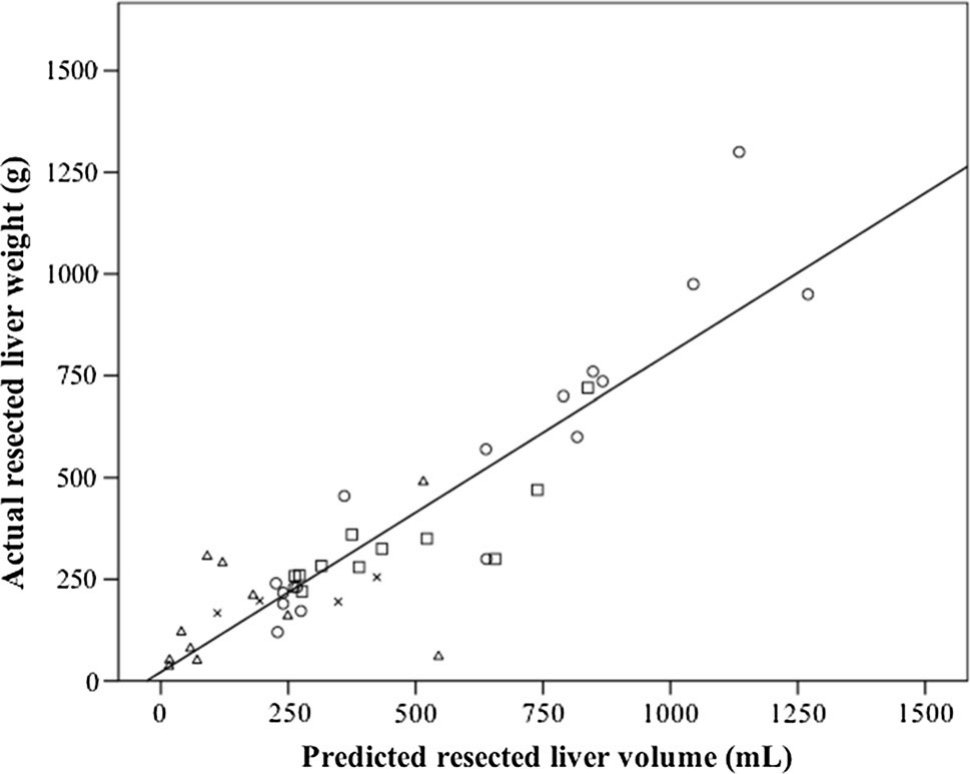
Correlation between the predicted resected liver volume and the actual resected liver weight. The simulated resection liver volume was compared with the weight of the actual resected liver specimen. Here, 1 g of liver tissue was presumed to correspond to a volume of 1 mL. The median absolute error between the predicted liver volume and actual liver volume was 88.6 mL (33.6%) and showed a strong correlation (*r* = 0.80, *P* < 0.001) with the actual resected liver volume. The* circles* represent hemihepatectomies or more complicated procedures. The* squares* represent sectionectomies. The* cross marks* represent segmentectomies, and the* triangles* represent partial resections

The correlation between predicted liver volume and actual resected liver weight is presented in Fig. 4. The median absolute error between the predicted liver volume and actual liver volume was 88.6 mL (33.6%), and this value indicated a strong correlation (*r* = 0.80, *P* < 0.001) with the actual resected liver volume.

## Discussion

The benefits of 3D simulation for liver surgery, such as an enhanced understanding of tumor anatomy, estimation of liver volume and assessment of irrigation area, have been previously reported [1, 2, 6–11]. However, no report has evaluated the influence of 3D simulation on perioperative outcomes [2]. The results of the present study suggest that 3D simulation may reduce operation time, especially for repeated hepatectomy and segmentectomy.

The complexity and variability of hepatic vasculature are responsible for the technical difficulty of liver surgery. For a surgery to be safe and effective, the positional and spatial relation between the tumorous lesion and blood vessels must be understood. However, sharing knowledge of an individual liver’s anatomy and resection line among the entire surgical team is difficult due to the necessity of reconstructing a 3D CT image in each physician’s mind. Recently, 3D simulation software has been developed to address the substantial quantity of imaging data acquired by advanced imaging modalities [12–15]. Therefore, we are now able to share a precise 3D surgical image preoperatively with ease. Based on a simulation, surgeons can obtain precise information about the position of crucial anatomic landmarks that previously could not be visualized [16]. The fact that the operation time for the 3D group was shorter than that for the without 3D group, the operations of which were performed by more experienced surgeons than those in the 3D group, supports this hypothesis. These simulation methods also open new avenues for future surgeries. These systems can be used not only to facilitate intraoperative procedures, but also to rehearse surgical maneuvers preoperatively [17].

Anatomic segmentectomy and subsegmentectomy have been proposed to increase the efficacy of surgical treatment for HCC [18, 19]. Generally, resection lines in hemihepatectomy and sectionectomy that are divided by hepatic veins are straight and easily recognized by demarcation lines or using intraoperative US. In contrast, compared with hemihepatectomy or sectionectomy, anatomic segmentectomy or partial resection can be more complicated to plan and perform. In the case of anatomic segmentectomy and subsegmentectomy, surgery requires technically demanding components, including the revelation of segment borders on the liver surface and liver parenchymal resection to expose hepatic venous tributaries with the assistance of intraoperative US [12, 18, 19]. Intraoperative US is currently the most useful modality used during liver surgery. However, serial intraoperative US images must currently be reconstructed in each surgeon’s mind based on the surgeon’s expertise [20]. Furthermore, a puncture and staining technique is helpful for recognizing each segment, but this technique requires additional operation time and demands a certain level of puncture skill [21]. Therefore, surgical experience and a high level of expertise are required for anatomic segmentectomy and subsegmentectomy. Similarly, in the case of repeated hepatectomy, it is difficult to recognize the modified anatomy, and the resection line of the liver can be complicated. Accordingly, we surmise that 3D simulation reduced operation time, especially for repeated hepatectomy and segmentectomy because it aided in planning surgical procedures and rehearsing maneuvers preoperatively. A 3D simulation system for anatomic repeated hepatectomy and segmentectomy provides a better understanding of the orientation of the liver structures and tumors, regardless of the surgeon’s expertise.

A small remnant liver volume is an important risk factor for postoperative liver failure, and assessment by CT volumetry using image analysis software is necessary to prevent postoperative liver failure. Previous studies have reported results similar to our findings, indicating that 3D simulation yields a precise liver volume prior to operation [6–9]. Precise anatomic resection may minimize peak AST level due to the decreased area of the devascularized remnant liver, and an accurate prediction of the remnant liver volume is believed to reduce the risk of postoperative liver failure. However, maximum AST level and postoperative liver failure tended to be worse in the 3D group in this study. We discuss 2 possible explanations for these results. The first explanation is a possible pitfall in the 3D simulation, for example, an error in anatomic reconstruction or overestimation of the remnant liver function. In the present study, the median absolute error was 33.6%, and the correlation (*r* = 0.8) was inferior to previous results (*r* = 0.94–0.995) [6–9]. The results indicate that the actual resection line differed from the planned resection line. Excess resection over the predicted volume would cause patients to experience liver failure. Another possible explanation is the expansion of surgical indication. The amount of blood loss, the incidence of bile duct leakage and other complications, except for liver failure, appeared to be similar between the two groups. This may be because the 3D group included more patients who were considered borderline resectable than the without 3D group. Since our study is retrospective, we could not assess all patients who were candidates for surgery. However, Radtke et al. [22] compared the resectability and operative strategy as determined by 2D CT imaging and 3D CT planning. They reported that 33% of the initial 2D resection plan was changed by 3D CT planning. Overall, 3D simulation facilitates the assessment of appropriate indications of surgery, PVE or other therapeutic options and allows borderline patients believed to be unrespectable by the conventional assessment to undergo surgery.

In this research, we focused on perioperative outcomes. However, 3D simulation has three other potential benefits. The first is a deeper understanding of the liver’s anatomy. Majno et al. [23] noted that for accurate liver surgery, it is necessary to understand the liver’s anatomy at a deeper level, such as the “1–2–20 concept”. This advanced level of anatomy is easily understood visually via 3D simulation. The second is the assessment of the irrigation and drainage area [22]. Congestion is an important problem in procedures that sacrifice major hepatic veins. Our colleague has previously reported the usefulness of 3D simulation for evaluating the drainage area [10]. The third is tailored anatomic liver resection. As Takamoto et al. [12] reported, 3D simulation facilitates the performance of segmentectomy and subsegmentectomy. These above benefits of 3D simulation would allow patients to undergo tailored anatomic liver resection with safe margins while saving liver function, leading to improved long-term survival.

Several limitations of this study should be addressed. Certain small vessel branches may have been missed by SYNAPSE VINCENT because not all of the vessels could be visualized by CT for each patient. This study was retrospective; therefore, several biases could not be excluded. A randomized prospective study must be conducted in the future.

## Conclusion

We demonstrate that the 3D preoperative simulation of liver surgery may reduce the operation time, especially for repeated hepatectomy and segmentectomy.
